# Survey Response Rates to a Self-Initiated Longitudinal Survey Accessed by a Quick Response Code in Six Different Regions of the United States

**DOI:** 10.7759/cureus.25146

**Published:** 2022-05-19

**Authors:** Lloyd M Halpern, De-An Zhang, Abby Velarde

**Affiliations:** 1 Pediatric Anesthesia, Shriners Children’s, Spokane, USA; 2 Pediatric Anesthesia, Shriners Children’s, Pasadena, USA; 3 Medical School, Washington State University, Spokane, USA

**Keywords:** orthopedics, pediatrics, response rates, survey response, qr code

## Abstract

Background

A quick response (QR) code allows rapid access to an online survey via a smartphone and may improve response rates for web-based surveys. We report the response rates for a QR code-based, self-initiated, longitudinal survey of opioid use and pain scores following hospital discharge in pediatric surgical patients.

Methodology

All parents of pediatric patients who underwent surgery at one of six pediatric medical facilities were asked to participate in the study from October 5, 2020, until July 15, 2021. Those who chose to participate accessed the initial enrollment survey using a QR code on a handout provided. The next day they received an emailed link to a daily survey until their child was not requiring opioids and had pain scores of less than 4 for the previous 48 hours.

Results

A total of 1,759 families were asked to participate in the study. The parents of 44 patients completed the initial enrollment survey by accessing the QR code (response rate of 2.5%). Of those who completed the initial survey, 67% were lost to follow-up during the survey series.

Conclusions

We found an extremely low response rate for a self-initiated survey accessed by QR code. Additionally, we found a drop in the response rate with each successive daily email-based survey. At the end of the survey series, the majority of the initial participants had dropped out. We recommend using alternative modalities (informed consent, telephone call, weekly surveys) for initiating and delivering surveys to improve response rates for similarly designed studies.

## Introduction

Survey response rates have declined dramatically in the past three decades. The response rate dropped from 67% in 1995 to 29% in 2018 in a British postal survey of new mothers who delivered in hospitals [1]. Surveyors continue to evaluate new survey modalities that may improve response rates. Web-based surveys can reduce the costs and time required of survey personnel. However, the majority of survey studies have reported reduced response rates for web-based surveys compared to surveys distributed by mail or telephone [2,3]. A quick response (QR) code allows rapid access to a web-based survey with a smartphone and may improve response rates for web-based surveys [4]. There is little information on the effect of alternative survey modalities on survey response rates. We are not aware of any reports of survey response rates associated with QR code access to a self-initiated, self-administered longitudinal survey. The primary aim of this study is to report the response rates of parents of recently discharged post-surgical pediatric patients to a self-initiated survey accessed by QR code in six cities in the United States. This article was previously posted as a preprint on the Research Square server.

## Materials and methods

All methods were performed in accordance with the relevant guidelines and regulations, as approved by the Washington Institutional Review Board (IRB) on 12/07/2020 (IRB tracking number 20201412). Following IRB approval, parents of pediatric surgical patients were asked to participate in the study from October 05, 2020, until July 15, 2021. All children under 18 years of age having surgery at six pediatric medical facilities were eligible to participate in the study (Lexington, Kentucky; Honolulu, Hawaii; Shreveport, Louisiana; St. Louis, Missouri; Pasadena, California; and Spokane, Washington). The number of eligible participants during this time period was released by each site without further disclosure of any Protected Health Information.

Due to limited research-trained staff, written consent was deferred at the time of the study introduction. Rather, the nurse or anesthesiologist caring for the patient provided parents with a brief verbal introduction to the study and an explanation of how to use QR codes. Parents were then given a handout containing a QR code, a Uniform Resource Locator (URL) link, and a detailed explanation of the study (Appendix 1). Those who chose to participate accessed the initial Enrollment Survey using the QR code. This survey presented information about the study and asked parents to provide an attestation that they had read and agreed to participate in the study. Parents who did not understand English or Spanish or did not have internet access were excluded from the study. All surveys were designed on the survey platform Qualtrics (Seattle, WA). All study materials, including the surveys, were available in Spanish and English.

**Figure 1:**
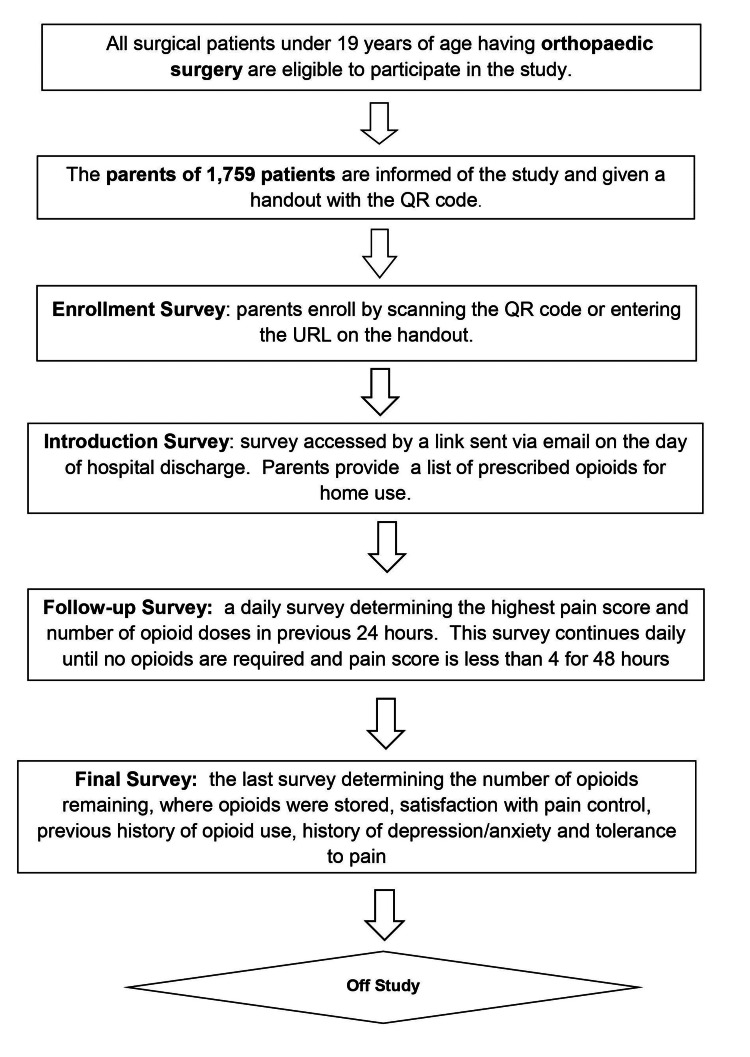
Study schema. QR: quick response

Our study schema is presented in Figure 1. During the study, parents received four types of surveys. The Enrollment Survey collected demographic data: the child’s birthdate, the surgery date, and an email address to receive links to subsequent Health Insurance Portability and Accountability Act (HIPAA)-compliant surveys. The Introduction Survey was sent on the day of hospital discharge determining any pain medications prescribed, including opioids and non-opioids. Next came the Follow-up Survey which asked for the highest pain score (0-10) and the number of opioids taken in the previous 24 hours. Parents received this until the child had not required opioids and reported a pain score of less than 4 for the previous 48 hours. Lastly, the Final Survey was sent asking how opioids were stored, the number of remaining opioids, if unused opioids were disposed of, if any non-opioid pain medications were used, side effects experienced from the pain medications, and parent’s satisfaction with their child’s pain control. The Final Survey also asked a series of questions regarding the child’s previous history with opioids, diagnoses of anxiety or depression, and the parent’s opinion of their child’s pain tolerance. The total number of surveys a parent received varied as surveys were designed to continue until no opioids were required and the pain was well controlled. The minimum time to complete the study was four days after surgery.

On April 12, 2021, an error occurred in the survey platform resulting in participants receiving no subsequent surveys after completing the Enrollment Survey. The study was terminated on July 15, 2021, because of an inadequate survey response rate.

The primary outcome of this report is the response rate to the initial Enrollment Survey by QR code. Secondary outcomes include the number of participants who were lost at each successive survey, mean time to complete each survey, survey completion rate, and the item response rate for completed surveys.

## Results

A total of 1,759 families were asked to participate in the study. Only 44 parents accessed the Enrollment Survey using the QR code (response rate of 2.5%). The response rate of individual cities is shown in Table 1. The range of response rates was 0.7% in Pasadena to 3.2% in Spokane, with all sites other than Spokane being under 2%. Overall, 40% (21/52) of the respondents were from the Spokane site.

**Table 1:** Response rate by site for initial survey accessed by QR code. QR: quick response

City	Number of patients	Number of respondents	Response rate (%)
Honolulu, Hawaii	183	2	1.1
Lexington, Kentucky	180	2	1.1
Pasadena, California	136	1	0.7
Shreveport, Louisiana	360	4	1.1
Spokane, Washington	660	29	4.4
St. Louis, Missouri	240	4	1.7
Total	1,759	52	2.96

The percentage of participants who were lost at each successive survey is displayed in Table 2. This analysis only includes patients who responded prior to the platform error. Here, 1,379 families were asked to participate and 33 enrolled. There was a loss of participants with each successive survey. Of those 33 enrolled, 19 (67%) failed to complete the longitudinal study leaving an overall study completion rate of 1% (14/1,379).

**Table 2:** Loss of participants with each successive survey.

Survey name	Enrollment survey	First sent survey	Follow-up survey	Opioid repeat survey	Final survey
Responses	33	25	20	15	14
Number lost	8	5	5	5	1
% lost	24	20	25	33	6

The number of questions for each survey, the mean time for survey completion, survey completion rate, and the item response rate are listed in Table 3. Of the 52 parents who used a QR code to access the Enrollment Survey, only 44 (85%) completed the survey. All parents who accessed subsequent surveys completed that survey. There were no skipped questions in any of the surveys. All surveys were brief, with the longest having 16 questions (Final Survey), and the longest mean time for completion of any survey was five minutes and five seconds (Enrollment Survey, including attestation). There were no skipped questions in any of the surveys.

**Table 3:** The number of questions, mean duration for survey completion, survey completion rate, and the item response rate for each survey.

Survey name	Enrollment	First sent	Follow-up	Opioid repeat	Final
Number of questions	10	7	3	2	16
Mean time (minutes:seconds)	5:05	1:32	0:14	0:25	2:49
Completion rate (%)	85	100	100	100	100
Item response rate (%)	100	100	100	100	100

## Discussion

The important findings of this study are an extremely low response rate (2.5%) for a self-initiated longitudinal survey accessed by QR code in six cities in the United States. We found a drop in the response rate with each successive email-based survey and the loss of the majority (67%) of the initial participants by the end of the survey series. These results were comparable in each participating region of the country.

The use of the QR code to access a survey by smartphone was first described in 2016 and offers promise as a new survey modality [5]. Eighty-five percent of American adults own a smartphone [6]. The use of the QR code reduces many of the problems associated with web-based surveys by immediately directing the person to the correct survey via the smartphone camera. However, there are limited reports of the use of the QR code to self-initiate a survey, with all involving small, targeted populations such as a class of medical students or patients in an urgent care waiting room [7,8]. This is the first report we are aware of evaluating the use of a QR code for self-initiated survey distribution in a large study population. In addition, we are unaware of a report demonstrating the response rate decline in a daily, longitudinal survey of any modality.

There are conflicting views on what is an adequate survey response rate. Federal public policy research surveys require an 80% response rate for funding. Editors of journals in the social and health sciences defined the lowest acceptable survey response rate as 60% [9]. Pew polling agencies conduct public opinion polls with accurate results with response rates in the single digits [10]. The concern about surveys with low response rates is that they may produce non-response bias, so that the results may not represent the intended population. More recent evidence suggests that, above a low threshold response rate, there is a weak relationship between the response rate and the non-response bias [11]. Although the absolute value of this low threshold is not defined, the 3% response rate we found likely falls below that threshold.

The reasons for the decline in survey response rates observed in the past two decades are many and likely influenced our response rate. One reason is increasing survey fatigue. In addition to our study, all our participants were also given a tablet to complete a survey while waiting for their first clinic appointment after surgery and many were approached to participate in a genomics study at their first clinic visit prior to surgery that includes a long survey of over 100 questions. Other reasons for declining survey response rates include concerns about the dissemination of personal information. This may have been an issue in our patient population as parents may have been hesitant to divulge their child’s opioid use for fear of identifying their child as having a persistent opioid use problem. We tried to account for this by making the survey anonymous. Our parents may have also been overwhelmed with caring for their child after surgery and having their other children at home during the coronavirus disease 2019 pandemic lockdown period and required remote learning. The use of a web-based survey accessible by a smartphone has been shown to produce lower response rates in patients over 75 years of age [12]. Our participants were all parents of children under 19 with the majority being in their third and fourth decades of life.

We found a loss of participants with each successive survey. Longitudinal studies conducted over many years report a decrease in participation rates with each follow-up survey because of a loss of interest in the survey subject [13]. This may have been a factor in our study as some participants remained in the survey for up to three weeks. Long and complicated surveys may produce a low survey completion rate and a high dropout rate with successive surveys. However, the brief survey duration and excellent survey and item completion rates in all four surveys make it unlikely that the surveys were the cause of the low response rates and high dropout rate.

We included data on participants after the error with survey distribution to illustrate the issues that web-based surveys present. Considerable time must be dedicated to designing these web-based surveys (six months) and continued vigilance is required to ensure proper function. Proper selection of a survey vendor is also key. Our study platform was designed for business applications so we were limited in our functionality when adapting it for a healthcare longitudinal study. We recommend thorough and thoughtful planning of the survey process, then ensuring the chosen vendor is able to execute this process.

Short message service (SMS) text messaging has shown improved response rates compared to email messaging. The combination of email and SMS messaging has produced the best results [14]. This study was intended to use SMS text messaging but, because of technical issues with Qualtrics, we were forced to use email. Although this may have improved our results, it is unlikely to have reached an acceptable response rate.

Other survey modalities have been associated with improved response rates. In a similar study that surveyed adult opioid use postoperatively after hospital discharge, patients were required to give informed, written consent prior to hospital discharge and were reached by phone calls on a weekly basis for four weeks [12]. A comparison in response rates reveals dramatically improved results. They had a similarly sized patient population of 1,880 patients who were approached to be in the study and 705 provided written, informed consent (38% response rate vs. 2.5%). Of these, 134 were lost to follow-up at the first phone call (20% vs. 24%). They lost 10 additional patients over the next month (1.4% vs. 40%). They reported that 561 patients completed the study (29.8% vs. 1.0%). These response rates are far greater than what we observed and suggest that informed, written consent and phone call follow-up at weekly intervals produce far superior results.

This study has limitations in both survey design and data collection. In regards to survey design, we were unable to verbally consent families leading to a lost opportunity for a person-to-person introduction to the study. Additionally, our survey platform lacked the ability to use SMS communication or send reminder emails. Finally, we were unable to offer families any incentives to participate in the study. In regards to data collected for the families that did participate, we had little demographic data to compare those who enrolled versus our surgical population. We did not collect family input on survey implementation. Finally, there is a possibility more families were unable to enroll due to survey platform malfunction.

## Conclusions

We found an unacceptably low response rate for a self-initiated, longitudinal survey accessed by QR code in a large study population. We found a loss of participants with successive daily surveys and the loss of the majority of the participants by the end of the survey series. While we believe QR codes remain a viable option for web-based survey distribution, the failure of this survey study provides lessons for future researchers. Survey design considerations should include maximizing participant recruitment, an end-user-friendly interface, limited survey questions and survey frequency, adequate reminder and follow-up, and, most importantly, a capable survey platform.

## Figures and Tables

**Figure 2:**
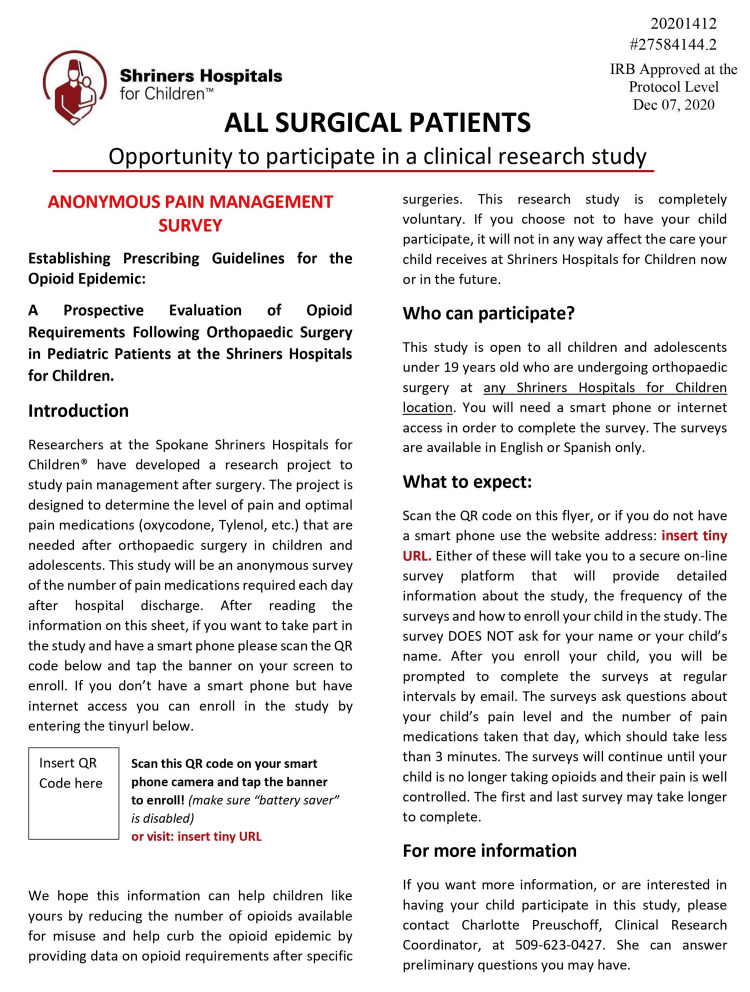
Survey handout with the quick response code.

**Figure 3:**
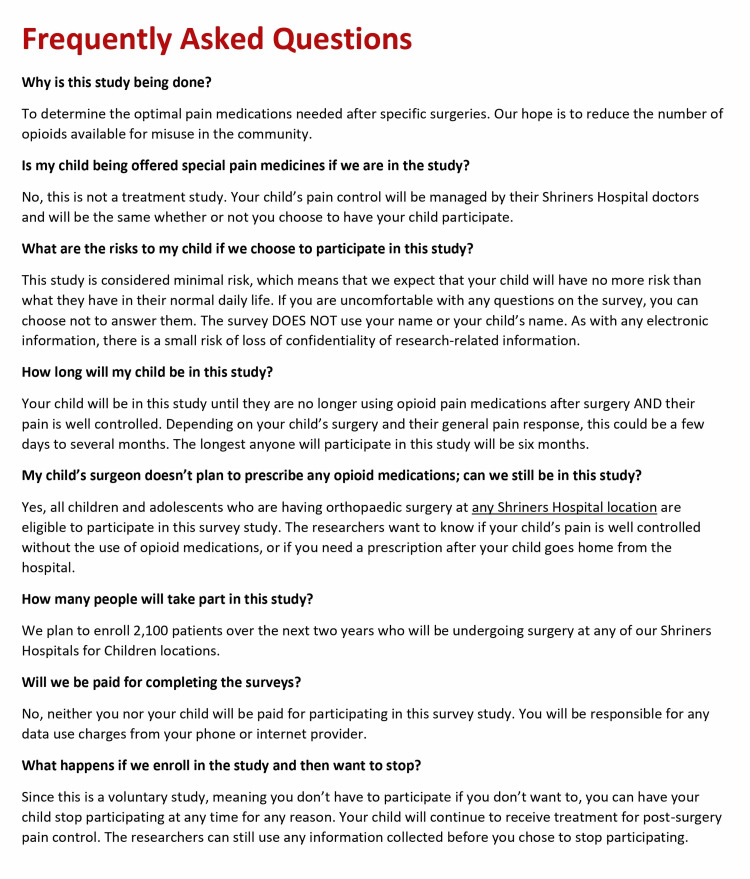
Page two of the survey handout with the quick response code.
